# The Correlation Between Migraine and Patent Foramen Ovale

**DOI:** 10.3389/fneur.2020.543485

**Published:** 2020-12-01

**Authors:** Kaiming Liu, Brian Z. Wang, Yishu Hao, Shuijiang Song, Mengxiong Pan

**Affiliations:** ^1^Department of Neurology of the Second Affiliated Hospital, Zhejiang University School of Medicine, Zhejiang University, Hangzhou, China; ^2^Nanyang Technological University Clinical Diagnostic Laboratory, NTU-Imperial Lee Kong Chian School of Medicine, Singapore, Singapore; ^3^Department of Neurology, Hangzhou Hospital of Traditional Chinese Medicine, Hangzhou, China; ^4^Department of Neurology, The First People's Hospital of Huzhou, Huzhou, China; ^5^Department of Neurology, The First Affiliated Hospital of Huzhou Teachers College, Huzhou, China

**Keywords:** migraine, patent foramen ovale, prevalence, patent foramen ovale closure, systematic review

## Abstract

**Background:** Migraine is a widespread neurological disorder. The patent foramen ovale (PFO) is a remnant of the fetal circulation. Multiple studies suggest that migraine is more prevalent in subjects with PFO and vice versa. It is unclear if there is a causal relationship or simply a co-existence of these two conditions. Furthermore, the treatment of migraine with percutaneous closure PFO remains controversial.

**Methods:** We reviewed studies pertaining to the relationship between PFO and migraine as well as the effects of treatments on migraine attacks.

**Results:** We briefly summarized potential pathophysiological mechanisms of migraine, and elaborated on migraine type, frequency, and clinical symptoms of migraine with PFO and the clinical features of PFO with migraine. We also addressed the effects of PFO closure on migraine attacks.

**Conclusion:** The evidence supports a “dose-response” relationship between migraine and PFO although more work needs to be done in terms of patient selection as well as the inclusion of an antiplatelet control group for PFO closure interventions to uncover possible beneficial results in clinical trials.

## Introduction

Migraine, one of the most common conditions of primary headache, often occurs in people aged 20-64 years old, with a high disability rate and heavy disease burden (1). According to the 2013 Global Burden of Disease survey from the World Health Organization (WHO), migraine was the 3^rd^ most common disease and ranked 6th in causing major disability in humans, which was calculated based on the number of years of life lost to disability (2).

The foramen ovale is a channel between the left and right atria of the heart during the embryonic period. Under normal physiological conditions, the foramen ovale will close in the first year after birth. If, however, it is not closed after three years of age, it is termed as patent foramen ovale (PFO) (3). It has been reported that PFO is the most common congenital cardiac anomaly in adults (4). In fact, about 20-30% of adults have an incomplete fusion of the fossa, which is a permanent slit-like interatrial opening (5). Usually, the blood pressure of the left atrium is higher than that of the right atrium, which will not cause right-to-left shunt (RLS). RLS via the PFO may occur when the pressure in the right atrium exceeds the left to give rise to structural changes in the heart, pulmonary hypertension, coughing, sneezing, and laughing. Although atrial septal defects and pulmonary arteriovenous malformations may also cause the right-to-left blood flow, they are relatively rare in migraine patients (6).

Del (7) first proposed the relationship between migraine and PFO in 1998, he found that the incidence of PFO in migraine patients was significantly higher than that in healthy controls. Later, a number of studies found that the incidence of PFO in migraine patients was 14.6-66.5% (8) while the incidence in the general population was 9-27.3% (5, 9, 10). In turn, in the population with PFO, the incidence of migraine was 9.13-51.7%, which was also higher than the incidence of migraine in the general population (11–13). However, until now, no consensus has been reached on the relationship between PFO and migraines. Therefore, this review aims to further investigate the association between migraine and PFO.

### Pathophysiological Mechanisms

The idea that migraine and PFO is correlated has only been around for a few decades, and much of the underlying pathophysiology is still based on hypotheses. It is thought that many vasoactive substances are usually discharged or metabolized through the pulmonary circulation. Through the PFO channel, venous blood can enter arterial blood by shunting without circulating in the lungs. Some chemicals and hormones such as serotonin can bypass the pulmonary circulation and pass directly through the blood-brain barrier to cause migraine (5). Moreover, a tiny embolus in the systemic circulation can pass through the PFO and directly into the arterial system. These “paradoxical embolisms,” which lead to tiny brain infarctions, triggering low perfusion or cortical spreading depression, may cause a migraine attack (5), and could be the most probable pathophysiological mechanism on how PFO could lead to a migraine attack. This hypothesis can also explain the use of antiplatelets or anticoagulants (14–16) and atrial fibrillation ablation (17) for relieving migraine attacks. Others have also found that a RLS is correlated with a higher frequency of multiple cortical lesions in DWI sequences, which distinguishes itself from atrial fibrillation-related ischemic stroke that is seen occurring in the cortical-subcortical territory (18). Incidentally, the posterior circulation is more likely to be involved (19). Blood flow of the posterior circulation significantly exceeds that of the anterior circulation in migraine patients with PFO when undergoing the Valsalva maneuver (20). During the aura phase, focal areas of hypoperfusion close to the ischemic threshold in occipital regions, which might be due to these cerebral microinfarcts, can cause visual symptoms (21). Meanwhile, a RLS results in decreased blood oxygen saturation and hypoxia, which increases the expression of plasminogen activator-1 and result in inhibition of fibrinolysis and thus increases the possibility of microembolization. On the other hand, a decrease in cerebral oxygen saturation will trigger cortical spreading depression as well, which can also lead to migraines (22, 23). Genetic factors may also cause these patients to develop both diseases. About 2-fold higher frequency of PFO is seen in migraineurs as compared with the general population, suggesting that a genetic influence could predispose some patients to a higher risk of developing both migraine and atrial septal abnormalities (4); hereditary associations with migraine have been found in autosomal dominant PFO (24). Taken together, the pathophysiological mechanisms are complex and migraine is possibly the result of these pathways working synergistically.

### Methods for Diagnosing PFO

Clinical examination methods are commonly used for diagnosing PFO including transesophageal echocardiography (TEE), contrast transcranial doppler echocardiography (cTCD) and contrast transthoracic echocardiography (cTTE). Among these, TEE is considered the gold standard for PFO diagnosis (7). However, due to the invasive nature of the procedure, patients find it difficult to successfully complete the Valsalva maneuver during TEE examination. Thus, the detection rate of RLS is lower than that of cTTE (8, 9), and have been shown to normally have a 10% rate of false negatives (10). cTCD is used to predict RLS by observing the amount of air microemboli in the cranial circulation at the resting state and after Valsalva maneuver. Although cTCD is a non-traumatic procedure, about 5% of shunts detected by cTCD does not correspond with PFO (11). The sensitivity and specificity of cTCD for RLS are 68-100% and 65-100%, respectively (12). Likewise, cTTE is also noninvasive but can isolate the source of RLS with a specificity of 97-100% albeit with a slightly lower sensitivity of about 63-100% (13, 14). At present, the varying diagnostic methods contributes to the diversity in the relationship between PFO and migraine. Therefore, a comparison of these individual methods and how it affects the relationship in question may be helpful.

## The Relationship Between Migraine and Patent Foramen Ovale

### The Relationship Between Migraine Type and Patent Foramen Ovale

The incidence of migraine with aura is 4.4%, comprising about 25-30% of migraineurs (25). Studies have found a stronger association between migraine with aura and PFO (26, 27). Among migraine patients, the incidence of PFO is 46.3-88% in migraine patients with aura (22, 23, 28, 29) compared with 16.2-34.9% in migraine patients without aura (30, 31). Interestingly, the incidence of PFO in migraine patients without aura is similar to that in the general population (6, 7, 9, 31). As PFO may be associated with migraine with aura, one study investigated the incidence of PFO in migraine patients with typical or atypical aura. The authors found that the PFO prevalence in the atypical aura group was 79.2% *vs*. 46.3% in the typical aura group (28). Therefore, it was suggested that PFO was more closely related to patients with atypical aura migraine but the specific mechanism remains unclear. Another report investigated the incidence of PFO in non-migraine patients with visual aura; 67% of the patients had PFO and 80% of those patients had improvement in symptoms after PFO closure, indicating that the presence of PFO could be one of the underlying mechanisms associated with aura pathology (32). Therefore, we consider the PFO having a closer relationship in migraine patients with aura, especially atypical aura, although non-migraine with aura is also correlated with the presence of the PFO.

### The Relationship Between Migraine Attack Frequency and Patent Foramen Ovale

Chronic migraine occur in about 2-3% of the population (33, 34). For one to be diagnosed with chronic migraine, the ICHD-3 criteria states that the patient would have “headache occurring on ≥15 days per month for >3 months, which has the features of migraine headache on ≥8 days per month” (35). Studies have shown that the incidence of PFO in chronic migraine, with aura or without aura, is higher. Of the 131 chronic migraine patients enrolled in a study, 66% had PFO, higher than PFO incidence in both the general population and episodic migraine patients (36). Another retrospective study focused on the relationship between visual aura frequency and PFO. A hundred and forty two migraine patients were divided into (i) frequent aura group (number of visual aura >50% of frequency of headaches) and (ii) accidental aura group (number of visual aura <50% of frequency of headaches). The results showed that migraine patients with frequent visual aura suffered a higher degree of RLS, and the symptoms improved after PFO closure (37). The high prevalence of PFO in chronic migraine patients do not indicate that PFO tend to stimulate chronic headaches, but still is associated with the number of migraine attacks, especially for large, high grade shunts.

### The Relationship Between Clinical Symptoms of Migraine and Patent Foramen Ovale

The clinical presentation of migraine seems indistinguishable in migraine patients with or without PFO. There is little statistically significant evidence in the patient's personal history, including age, sex, smoking history or migraine onset, including the symptoms of headache, and concomitant symptoms of PFO. The SAM (Shunt-Associated Migraine) study was a prospective, multicenter, observational study, intended to illustrate the difference of the clinical features of migraine with or without blood flow shunt. A total of 460 patients were included in the study. Migraine patients with RLS and without RLS accounted for 58% and 42% of the total patients, respectively. Migraine features were not significantly correlated, except that patients with RLS were relatively young and had aura sensory symptoms with slightly higher frequency (38). In chronic migraine patients, PFO and non-PFO patients have similar headache characteristics and neurological symptoms (36). PFO seems to play a role in triggering migraines but have little relation to migraine symptoms. Recently, it was found that the attack frequency, HIT-6, and MIDAS scores among migraine patients with moderate or large PFO were significantly higher than those of the mild PFO and non-PFO groups. After PFO closure, the differences in VAS, HIT-6 and MIDAS scores as well as the headache duration were statistically significant (39). At this point in time, it cannot be concluded that the scale scores changed due to the attack frequency or severity, but the result does provide more evidence supporting the relationship between PFO presence and migraine presentation. Studies are now needed to explore the correlation and the analysis of the scale needs to be refined.

## The Relationship Between Patent Foramen Ovale and Migraine

### The Relationship Between the State of Patent Foramen Ovale and Migraine

Under resting conditions, no RLS exists generally because the blood pressure in the left atrium is higher than in the right. After performing the Valsalva maneuver, the pressure of the right atrium will exceed that of the left atrium to give rise to transient RLS. During PFO examination, the RLS should be detected at rest and post-Valsalva. RLS occurring under normal respiration is called permanent PFO while RLS occurring only after the Valsalva maneuver is called latent PFO (23).

Persistent shunt accounts for 67-72% and latent shunt exist 28-33% in migraine with PFO patients (22, 23, 29). One study showed that 12 of the 159 migraine patients with aura experienced a migraine attack when they were undergoing the cTCD test. Surprisingly, all these patients had permanent PFO and the majority were massive shunts (22), indicating that permanent PFO is closely associated with migraines and triggers a migraine attack.

### The Relationship Between the Size of Patent Foramen Ovale and Migraine

PFO is usually divided into three types: large PFO (≥4.0 mm), medium PFO (2.0-3.9 mm) and small PFO (≤1.9 mm) (40). However, this classification standard is only accurately measured by autopsy or estimated by TEE, which is not commonly used in clinical practice. RLS from PFO is considered when microvesicles are found within 3-5 cardiac cycles during a cTTE examination (41, 42). PFO size is usually graded according to the number of microbubbles in the left atrium on a single still image. cTCD is graded according to the number of microvesicles found in the bilateral cerebral circulation. The amount of RLS detected by cTCD is positively correlated with the size of PFO measured by TEE (43).

Approximately 75% of migraine patients with PFO have a large RLS and 25% have a small shunt (23). Among all PFO subjects, the proportion of large triage is higher in migraine patients than in healthy subjects. In migraine patients with aura especially, a greater proportion of permanent PFO and large PFO were found (44). Schwartzman (6) studied 93 migraine patients with aura and 93 healthy controls. All subjects underwent cTTE and they found that the number of people having small RLS among migraineurs and healthy controls were similar but a moderate or large RLS occurred more frequently in the migraine group. Similarly, among patients with cryptogenic stroke, Anzola (45) found that migraine patients had a larger shunt *vs*. non-migraine patients. The difference was even more pronounced when compared with the control group. PFO is also considered a probable risk factor in cryptogenic stroke of which micro-embolism may contribute to its pathogenesis. PFO is frequently found in older patients with stroke (46) as well as several other stroke subtypes (47, 48), and the stroke attributable fraction for PFO can be defined through standardized scores (48, 49). Taken together, PFO may be associated with migraine and could increase the risk of stroke in migraine patients. The larger the PFO size indicates a larger RLS, which is more likely to cause a migraine.

### The Relationship Between Patent Foramen Ovale Anatomical Structures and Migraine

Atrial septal aneurysm (ASA) is a kind of congenital atrial septal dilatation, which means that the atrial septal distention is >10 mm in one side of the atrial septal plane, and the basal width of the tumor is >15 mm, involving the fossa ovalis region (3). According to echocardiography studies and post-mortem epidemiological studies, the prevalence of ASA of the general population is about 1-2.5% (9, 50). PFO complicated with atrial septal tumor have been shown to be a risk factor for both cryptogenic (51, 52) and recurrent cryptogenic strokes, suggesting that medical treatment could be refined (53–55). Recent studies also showed that migraine has a high correlation with PFO and ASA (44, 56). In Snijder's study (44), the prevalence of PFO with ASA was significantly higher in migraine patients compared to patients without migraine. In addition, the shunt of patients with PFO combined with ASA was significantly larger than that of patients with PFO alone. Therefore, the combination of PFO and ASA may lead to increasing shunt flow and the occurrence of migraine.

The Eustachian valve (EV) and Chiari network (CN) are remnants of venous valves caused by incomplete absorption of these structures (52, 57). Embryologically, EV is a semicircular structure facing the anterior-inferior aspect of the inferior vena cava, directing blood flow from the inferior vena cava to the fossa ovale, which plays a vital role in the shunt of blood flow to the ovale (58, 59). The CN is a large multi-perforated EV with a reticular appearance found in approximately 2% of the population (60). Previous studies had reported that EV and CN were more common in patients with cryptogenic stroke (61, 62). Rigatelli (63) prospectively investigated the potential effects of EV/CN on migraine patients with PFO and found that the frequency of EV/CN was 100% and 60% in migraineurs with aura and migraineurs without aura, respectively. Meanwhile, patients with EV/CN had more “curtain pattern,” larger RLS on TCD and higher preoperative MIDAS score. After PFO closure, the MIDAS score decreased significantly. Formation of an atrial septal aneurysm and persistent EV/CN may prevent spontaneous closure of PFO afterbirth, facilitating RLS, and indirectly inducing a migraine attack.

### Treatment of Migraine With Foramen Ovale Closure

Migraine and PFO may be in a “dose-response” relationship. For example, the large size, persistency of PFO, and anatomic variations of PFO can intensify RLS, by which increased vasoactive substances or tiny emboli can pass through the blood-brain barrier and cause a higher number of hypoperfusion events to result in a migraine. These provide further evidence for migraine treatment, not only more accurate treatment choices for different patients, but also help guide surgical treatment.

In 1992, percutaneous PFO closure was first performed (64), and the benefits of PFO closure in migraine patients were first reported in 2000 (65). Since then, there have been a series of studies about the effect of PFO closure in migraine. Among the case series studies, PFO closing resolved headaches in 14-85% of patients, among which 25-85% had migraine with aura and 14-50% were migraine without aura. 4-58% of patients had ameliorated migraine with aura and 20-68% migraine without aura. 6-43% of patients had no change in symptoms while 3-8% had worse symptoms (65–85) (see Table 1). In the case control studies, PFO closure is also associated with decreased migraine severity (32, 86–90) (see Table 2). In a study, PFO closure had statistically significant benefit with VAS, HIT-6 and MIDAS scores and the headache duration (39), especially for patients younger than 45 years (91). In addition, the closure of PFO resulted in a significant reduction in the use of abortive medications (86).

**Table 1:** The Effect of Patent Foramen Ovale Closure on Migraine in case series studies.

**References**	**Type**	**Sample size**	**Age**	**Diagnostic mode**	**Residual shunt**	**Resolved**	**Improved**	**No change**	**Worsened**	**Follow-up (months)**	**Antiplatelet therapy time (months)**	**Adverse events**
Wilmshurst et al. (65)	Retrospective	21	38.2	cTTE	0	10 (48%)	8 (38%)	3 (14%)	0	9-32	6	
Morandi et al. (66)	Prospective	17	48 ± 15	cTCD	4 (24%)	5 (29%)	10 (59%)	2 (12%)	0	6	6	AF in 2 subjects
Schwerzmann et al. (67)	Retrospective	MA 37	49 ± 11	TEE	3 (8%)	4 (11%)	26 (70%)	7 (19%)	0	20.4 ± 10.8	6	
		MO 11	42 ± 12			1 (9%)	9 (82%)	1 (9%)	0			
Reisman et al. (71)	Retrospective	MA 39	47 ± 12	cTCD and/or TEE	14 (72%)	21 (54%)	5 (14%)	12 (32%)	0	9.25 ± 5.75	6	
		MO 18				7 (62%)	2 (15%)	3 (23%)	0			
Azarbal et al. (68)	Retrospective	MA 24	49 ± 13	TEE	12 (18%)	18 (75%)	1 (4%)	-	-	12	-	
		MO 13				4 (31%)	5 (38%)	-	-			
Ferrarini et al. (69)	Retrospective	5	40.2 ± 11.3	cTCD, TEE	1 (20%)	4 (80%)	1 (20%)	0	0	6	-	
Mortelmans et al. (70)	Retrospective	MA 8	47 ± 13	-	-	4 (50%)	-	-	-	29	-	10 patients who did not have migraine before developed migraine
		MO 14	35 ± 14			6 (43%)	-	-	-			
Giardini et al. (72)	Prospective	MA 13	43 ± 13	TEE	6 (16%)	11 (84%)	1 (8%)	0	1 (8%)	58.5 ± 16.8	12	
Giardini et al. (73)	Retrospective	MA 35	41.1 ± 11.0	TEE	6 (17%)	29 (83%)	3 (8%)	2 (6%)	1 (3%)	20.8 ± 16.3	12	
Dubiel et al. (74)	Retrospective	MA 24	44 ± 13.5	TEE	1 (2.2%)	8 (33%)	14 (58%)	2 (8.3%)	0	39.6 ± 23.9	6	
		MO 22				3 (14%)	15 (68%)	4 (18.2%)	0			
Jesurum et al. (75)	Retrospective	MA 55	47 ± 12	cTCD and/or TTE	23 (34%)	36 (54%)	17 (25%)	11 (16%)	3 (5%)	18	6	
		MO 22	46 ± 10									
Luermans et al. (76)	Prospective	MA 10	51.6 ± 12.3	TEE	-	8 (80%)	-	-	-	6	6	1 TIA/1 ischemic stroke/1 inguinal hematoma/1 did not unfold
		MO 14				7 (50%)	-	-	-			
Chessa et al. (77)	Prospective	MA 28	40.2 ± 11.2	TEE	10 (23.8%)	7 (25%)	14 (50%)	-	-	6	6	
		MO 14	21 ± 11.2			4 (29%)	8 (57%)	-	-			
Wahl et al. (79)	Retrospective	MA 14	44 ± 12	TEE	1 (6%)	4 (29%)	4 (29%)	6 (43%)	0	32.4 ± 18	5	
		MO 3				0	2 (67%)	1 (33%)	0			
Papa et al. (79)	Prospective	76	43.2	TEE	2 (2.6%)	35 (46%)	27 (36%)	14 (18%)	0	13.7 ± 2.4	6	5 inguinal hematomas/1 AF
Wahl et al. (81)	Retrospective	MA 96	51 ± 11	TEE	14 (9%)	37 (39%)	44 (46%)	8 (8%)	7 (7%)	60.0 ± 22.8	-	1 TIA and 1 ischemic stroke
		MO 54	53 ± 11			14 (26%)	23 (42%)	15 (28%)	2 (4%)			
Rigatelli et al., (80)	Prospective	MA 34	40 ± 3.7	TEE, cTCD	2 (6%)	19 (56%)	6 (18%)	2 (6%)	-	9.0 ± 2.8 m	-	AF in 3 subjects
Trabattoni et al. (82)	Prospective	77	42.6 ± 12	TEE, cTCD and cTTE	-	26 (34%)	46 (60%)	5 (6%)	0	28 ± 27	-	Transient AF 15/vascular complications 2/Device malpositioning 1
Rigatelli et al. (83)	Prospective	80	42 ± 2.7	TEE, cTCD	7 (8.7%)	41 (51%)	21 (26%)	18 (23%)	0	50.1 ± 16.8	-	AF 3
Araszkiewicz et al. (84)	Prospective	MA 4	38 ± 18	TEE, cTTE	-	2 (50%)	-	-	-	28.6 ± 12.1	6	Supraventricular arrhythmias 3, groin hematoma 3, neurological events 5
		MO 17				8 (47%)	-	-	-			
Milev et al. (85)	-	22	40.7 ± 11.7 years	cTCD, TEE	15 (42.9%)	4 (18%)	18 (82%)	0	0	24	6	One femoral hematoma and hypotension; one chest pain

*MA, migraine with aura; MO, migraine without aura; AF, atrial fibrillation; TIA, transient ischemic attack; “-”: not mention*.

**Table 2:** The Effect of Patent Foramen Ovale Closure on Migraine in case-control studies.

**References**	**Type**		**Sample size**	**Age**	**Diagnostic mode**	**Occluder devices**	**Residual shunt**	**Resolved**	**Improved**	**No change**	**Worsened**	**Follow-up**	**Antiplatelet therapy time (months)**	**Adverse events**
Kimmelstiel et al. (86)	R	Closure	24	63	TEE	Amplatzer	3 (13%)		20 (83%)	2 (8%)	2 (8%)	3	-	
		Open	26	62					0	25 (96%)	1 (4%)			
		Control	10	54					1 (10%)	9 (90%)	0			
Vigna et al. (87)	P	Closure	53		cTCD, TEE	Amplatzer/Cardia/ CardioSEAL/STARFlex	3 (6%)	18 (34%)	28 (53%)	7 (13%)	0	16 ± 7	6	-
		Control	29					2 (7%)	5 (17%)	19 (66%)	3 (10%)			
Rigatelli et al. (88)	P	Closure	MA 32	35 ± 6.7	cTCD, TEE	Amplatzer/Premere	2 (5%)	15 (47%)	12 (38%)	5 (16%)	0	29.2 ± 14.8	-	AF 2
			MO 8					2 (25%)	4 (50%)	2 (25%)	0			
		Control	MA 10					0	0	10 (100%)	0			
			MO 36					0	0	36 (100%)	0			
Khessali et al. (32)	-	Closure	MA72	48 ± 13	cTCD, TEE	CardioSEAL/Amplatzer/ Helex	-	39 (54%)	16 (22%)	1 (1%)	3 (4%)	12	-	-
			MO 8					6 (75%)	0	1 (13%)	0			
		Control	36	54 ± 17				-	-	-	-			
Biasco et al. (89)	R	Closure	MA 67	46.4 ± 12.7	TEE, cTCD	Amplatzer/Cardia/ Others	16 (24%)	36 (54%)	18 (27%)	10 (15%)	3 (5%)	46.6 ± 32.7	6	One endocarditis
			MO 22					10 (45%)	10 (45%)	0	2 (9%)			
		Medical	MA 82	47.1 ± 12.3				20 (24%)	19 (23%)	35 (43%)	8 (10%)			
			MO 46					11 (24%)	16 (35%)	14 (30%)	5 (11%)			
Xing et al. (90)	-	Closure	125	39.0 ± 12.9	cTCD, cTTE	Cardi-O-Fix	6 (5%)	67 (53.6%)	92 (76.3%)	31 (24.8%)	2 (1.6%)	12	6	One cardiac tamponade

*MA, migraine with aura; MO, migraine without aura; AF, atrial fibrillation; “-”: not mention; “r”: retrospective; “p”:prospective*.

However, in randomized controlled studies, the results were unremarkable (see Table 3). Migraine Intervention With STARFlex Technology (MIST) is the first prospective, multicenter, double-blind, controlled study to evaluate the efficacy of PFO with STARFlex implants for refractory migraine. After follow-up at 6 months, there were no significant differences (implant *vs*. control group) in the main therapeutic endpoints and headache cessation at 91-180 days after the closure. The same results were seen at the secondary endpoint. Upon further exploratory analysis, after excluding two outliers, the implant group saw a significant reduction in number of migraine days. With respect to the results of MIST and observational studies, it was explained that, first of all, there were physiological differences between the study group and the group of patients treated in the observational study. In addition, the RLS might not be effectively closed by the device used, which resulted in differences in experimental results (92). Subsequently, the PRIMA (Percutaneous closure of patent foramen ovale in migraine with aura) study also aimed to evaluate the efficacy of percutaneous PFO closure in patients of migraine with aura who were refractory to medical treatment. The primary and secondary efficacy endpoints, the decrease number of migraine attacked in 3 months after treatment and the average decrease number of migraine attacked separately, including the total cessation of headache, all improved in the treatment group, though with no statistical significance (93). Recently, PREMIUM (Prospective, Randomized Investigation to Evaluate Incidence of Headache Reduction in Subjects with Migraine and PFO Using the AMPLATZER PFO Occluder to Medical Management) studied patients with 6-14 days of migraine per month who had a large RLS and failed at least three preventive medications. For the primary efficacy end event endpoint, a 50% reduction in the number of headache episodes in the procedure group was seen though again, was not statistically significant. The secondary efficacy endpoint which was to reduce the number of headache days saw statistically significant differences in both groups. In the subgroup analysis, the proportion of frequent migraineurs with aura (more than 50% of migraine attacks with aura) reaching the primary efficacy end event was significantly higher than that of the control group, even 15.4% patients had headache cessation (94). These RCT results, although mostly insignificant, can still inform future clinical trials in terms of (i) patient selection e.g., patients with migraine with more frequent aura attacks and patients PFO with large RLS can be top priorities since these are affected by the “dose-response” relationship of migraine and PFO; and (ii) additional subgroup analyses e.g., excluding outliers. In fact, present ethical considerations stipulate that clinical studies can only recruit those with severe refractory migraine and not non-refractory migraine, which is an issue that needs to be addressed since a study design involving non-refractory migraine patients would be beneficial on many levels.

**Table 3:** The Effect of Patent Foramen Ovale Closure on Migraine in randomized controlled studies.

	**Subjects**	**Mean age**	**Diagnostic mode**	**Randomization**	**Follow-up**		**Result**				**Antiplatelet therapy**
						Primary endpoint	Secondary endpoints			Exploratory analysis	
MIST trial	MA with frequent attacks, failed ≥2 prophylactic treatments, moderate or large RLS with PFO		cTTE, TEE		180 days	Cessation of migraine headache	Frequency of attack reduction (days/month)	Total MIDAS score	Total HIT-6 score	Reduction in total migraine headache days (excluding 2 outliers)	Aspirin and clopidogrel were given 300 mg in the 24 h before the procedure and 75 mg each daily for 90 days after the procedure
Intervention group	74	44.3 ± 10.6		STARFlex septal repair implant		3	3.26 ± 1.82	16 (0–270)	60 ± 10		
Control group	73	44.6 ± 10.4		Sham procedure (skin incision in the groin)		3	3.55 ± 2.14	18 (0–240)	59 ± 8.8		
P						1	0.13	0.89	0.79	0.027	
										*Post-hoc* analyses	
PRIMA	Unresponsive to 2 preventive medications MA with PFO		cTTE or cTEE and TEE		1 year	Reduction migraine (days/month)	The average attacks reduction	≥50% reduction of migraine days	MIDAS score improvement	Mean reduction in MA (days/month)	Aspirin 75–100 mg/day for 6 months, clopidogrel 75 mg/day for 3 months
Intervention group	53	44.1 ± 10.7		Amplatzer PFO Occluder		−2.9	−2.1	15 (38%)	−18.3	−2.4	
Control group	54	42.7 ± 11.0		Medical management		−1.7	−1.3	6 (15%)	−13.9	−0.6	
P						0.17	0.97	0.0189	0.53	0.0141	
PREMIUM	Failed ≥3 preventive medications, 6–14 days/month migraine with RLS		cTCD, cTTE		1 year	50% reduction in attacks	Number of migraine (days/month)	≥75% reduction in migraine attacks	Complete cessation of migraine attacks		Pre-treated with aspirin 325 mg and clopidogrel 600 mg
Intervention group	117	42 ± 10		Amplatzer PFO Occluder		45 (38.5%)	−3.4 ± 4.4	24 (20.5%)	10 (8.5)%		
Control group	103	41 ± 10		sham procedure		33 (32%)	−2.0 ± 5.0	17 (16.5%)	1 (1%)		
P						0.32	0.025	0.45	0.01		

*MA, migraine with aura*.

Considering the abnormal coagulation mechanism, the formation of “micro-embolisms” may also be one of the important causes of migraine. Some studies had reported the effects of antiplatelet agents on PFO associated with migraine (95, 96). A study of 136 patients with migraine and PFO who had a stroke previously, found that 90 patients (66%) had ≥50% decreased headache days per month compared to baseline after administration of clopidogrel or prasugrel. Fifty-five patients received PFO closure and discontinued antiplatelet medication 3 months after PFO surgery. Out of these, 52 patients (94%) had relief of headaches up until the follow-up of 6 years. Twenty-six patients without PFO closure who had been taking antiplatelet drugs also responded favorably up till 4 years of follow-up. However, 8 patients who did not take antiplatelet drugs or receive PFO closure, later experienced headache after 4-5 days, which was the anticipated washout period for the antiplatelet drugs. Antiplatelet medicine and PFO closure had a similar effect in migraine patients, so it was speculated that migraine pathogenesis involves venous platelet activation or aggregation, wherein tiny emboli causes the migraine (97). Subsequently, a prospective study found that the use of Tigarelor also reduced the frequency of migraine attacks in some PFO patients (98). Therefore, postoperative antiplatelet drug should be considered an important confounding factor in assessing the efficacy of PFO closure, and future research should consider setting the antiplatelet medication group as the control group for PFO closure. Of note, many studies of follow-up periods of less than 6 months possibly had their results confounded by the administration of antiplatelet drugs which they did not take into account during analyses.

## Summary

In this review, we attempted to specifically address the relationship between migraine and PFO, elucidate mechanisms and improve estimation of the risks and benefits of the different therapeutic strategies available. The incidence of PFO in migraine patients is higher than that in the general population, suggesting that PFO and migraine may be risk factors for each other, but more research is needed to confirm this speculation. An increasing number of studies have found that migraines with aura are more closely associated with PFO, and the presence of RLS increased the likelihood of aura attacks, reducing the susceptibility to migraine attacks after exposure to other triggers. The frequency of headache onset, but not its clinical features, is also correlated with PFO, which seem to suggest that RLS may trigger the onset of migraine without directly affecting the migraine symptoms. In addition, the type and size of the foramen ovale are also associated with migraine. Persistent PFO, larger PFO, and complex tissue structures may cause more RLS of the blood to increase the incidence of migraine. Taken together, these support the “dose-response” relationship between RLS and migraine.

Based on the current findings, PFO occlusion was not satisfactory for the improvement of headaches in migraine patients. More accurate adequate patient recruitment may lead to greater postoperative benefit and more significant symptom improvement. Observational studies may further elaborate on the relationship between migraine and PFO type. Furthermore, randomized controlled studies should not be limited to patients with medication refractory migraineurs. Moving forward, investigation is needed to identify those migraineurs who are more likely to benefit or invalid from the closure of PFO, and in our opinion, migraines with more frequent aura attacks and PFO with larger RLS shunt should be research priorities. In addition, antiplatelet agents must be a control group for clinical trials of PFO closure. Lastly, researchers should consider that the closure of PFO may carry a small but relevant risk of serious adverse events including stroke, pericardial tamponade, atrial fibrillation and death (99).

## Author Contributions

KL conceived the idea for and edited the manuscript. BZW substantially revised the manuscript for readability and intellectual content. YH contributed to table design and manuscript revision. SS contributed expert medical advice and to manuscript revision. MP reviewed the literature and drafted and edited the manuscript. All authors read and approved the final submitted version.

## Conflict of Interest

The authors declare that the research was conducted in the absence of any commercial or financial relationships that could be construed as a potential conflict of interest.
